# Bootstrapping random forest and CHAID for prediction of white spot disease among shrimp farmers

**DOI:** 10.1038/s41598-022-25109-1

**Published:** 2022-12-03

**Authors:** Michael Onyema Edeh, Surjeet Dalal, Ibidun Christiana Obagbuwa, B. V. V. Siva Prasad, Shalini Zanzote Ninoria, Mohd Anas Wajid, Ademola Olusola Adesina

**Affiliations:** 1 Department of Vocational and Technical Education, Faculty of Education, Alex Ekwueme Federal University, Ndufu-Alike, Abakaliki, Nigeria; 2 Department of Mathematics and Computer Science, Coal City University, Enugu, Nigeria; 3 Amity University Haryana, Gurugram, 122413 India; 4 Sol Plaatje University, Kimberley, 8305 Northen Cape South Africa; 5 Department of CSE, School of Engineering, Malla Reddy University, Hyderabad, India; 6 College of Computing Science and IT, Teerthanker Mahaveer University, Moradabad, Uttar Pradesh India; 7 Department of Computer Science, Aligarh Muslim University, Aligarh, 202002 India; 8 Department of Mathematical Sciences, Olabisi Onabanjo Univeristy, Ago-Iwoye, Nigeria

**Keywords:** Computational biology and bioinformatics, Engineering, Diseases

## Abstract

Technology is playing an important role is healthcare particularly as it relates to disease prevention and detection. This is evident in the COVID-19 era as different technologies were deployed to test, detect and track patients and ensure COVID-19 protocol compliance. The White Spot Disease (WSD) is a very contagious disease caused by virus. It is widespread among shrimp farmers due to its mode of transmission and source. Considering the growing concern about the severity of the disease, this study provides a predictive model for diagnosis and detection of WSD among shrimp farmers using visualization and machine learning algorithms. The study made use of dataset from Mendeley repository. Machine learning algorithms; Random Forest classification and CHAID were applied for the study, while Python was used for implementation of algorithms and for visualization of results. The results achieved showed high prediction accuracy (98.28%) which is an indication of the suitability of the model for accurate prediction of the disease. The study would add to growing knowledge about use of technology to manage White Spot Disease among shrimp farmers and ensure real-time prediction during and post COVID-19.

## Introduction

Technology has become an indispensable tool in the health sector. Many researchers are leveraging the benefits of technology to develop health models and systems to improve health outcome ^1–4^. White Spot Disease (WSD) is a highly contagious viral illness of decapod crustaceans that has a high fatality rate in White Spot Disease shrimp produced in the laboratory. Since its first epidemic in 1992–1993, this illness has resulted in significant losses in the economy. Large, non-occluded DNA viruses with tail-like extensions at one end are the cause of white spot syndrome (WSD) and are known as white spot syndrome virus (WSSV). It grows in the nucleus and has a wide spectrum of hosts among crustaceans, making it particularly difficult to eradicate. In accordance with this, Bangladesh had to lose USD 20 billion last year from WSD. It is also challenging to get any convenient procedures within the farming practice so that the horizontal transmission can be diminished through satisfied treatment for vertical transmission as there are chances of occurrences in both vertical and horizontal transmission of White Spot Syndrome Virus (WSSV). With the help of detailed literature review, the past datasets havebeen analyzed and has provided proficient procedure about the shrimp farming system which practices in Bangladesh and also highlights the shortcomings in between the existing practices. It also recognizes probable WSD risk reduction procedures. A WSSV was recently reclassified as the solitary species in a new monotypic family named Nimaviridae based on genetic analysis and physical traits (genus Whispovirus).

In this research, we present an Ensemble learning model based on the Random Forest model and the CHAID model to detect the characteristics of White Spot Disease and provide an accurate and rapid diagnosis. This system has been constructed in three stages. First, imputation based approaches in machine learning have been used to do pre-processing tasks such managing missing values and outliers. The suggested approach attained an accuracy of 98.28% when feature extraction was used to get values of features associated with that illness and further feature classification was performed using a Random Forest classifier trained on CHAID afflicted and non-diseased fish.

The rest of this work is organized as follows: section“ Literature review” describes the details of the existing works; in section “Materials and methods”, the proposed methods are utilized to predict the whitespot disease; the results are being represented in section “Results” and finally, the conclusions are summarized.

## Literature review

Karim et al.^5^ was the first to raise awareness of the leukaemia sickness, and many others have contributed to this subject. Some of the most popular machine learning methods, including decision trees, naive bayes, Random Forests, gradient boosting machines, linear regression, support vector machines, and the ensemble LDSVM model, which is a mix of Logistic Regression (LR), DT and SVM, are shown. For the categorization of leukaemia in patients and the analysis of their effects, the k-fold cross-validation and grid search optimization approaches were often utilised with the LDSVM model. The study's goal was to accurately and quickly identify people who will develop leukaemia. The method used a more fair comparison to assess variables including precision, recall, and f1. Additionally, an SMOTE and PCA method were used to create a synthetic minority oversampling strategy. Fast and rapid experiment execution was developed using a newly-developed approach for decreasing the column results.

White spot syndrome virus (WSSV) causes white spot disease (WSD) in shrimp and many other crustaceans, according to Dey et al.^6^. Death occurs within 3–10 days of an outbreak under standard culture conditions because the virus is extremely infectious. From its initial breakout in China and Taiwan in 1991 and 1992, it can be deduced that the globe has had to endure exceptionally significant losses in agriculture. Many WSSV reports point to the need of advanced genetic investigations and biosecurity procedures in the fight against illness. Greenhouse, polyculture, biofloc and limited water exchange are some of the most recent WSD management systems that have emerged. In order to make the most of the latest technology, open research questions must be addressed. White spot syndrome virus was discovered in shrimp by Nagalakshmi et al.^4^ using the image processing approach K-Means clustering (Penaeid prawn). Biosensoris is one of the most sensitive capacitive methods for detecting WSSV in aquaculture methods, and it uses WSSV shrimp pond water and mixes glutathione-S-transferase tag for white spot binding protein (GST-WBP) and immobilises on a gold electrode through a self-assembled monolayer to detect WSSV. Capacitance measurement may be used to quickly discover the binding between WSSV and GST-WBP. The capacitive biosensor can detect shrimp species under ideal conditions. This procedure is more time-consuming than the others. Image segmentation techniques, on the other hand, can deliver more accurate findings in a shorter amount of time at a lower cost. In the suggested method, images are acquired and then processed using image sensing techniques such as histograms and K-Means clustering.

According to Adl et al.^7^, fish illness detection is a difficult categorization technique that needs expert knowledge at the specialist level. As a result, he developed a system that uses image processing, machine learning, and swarm optimization to accurately diagnose fish ailments. As a first step, the system does pre-processing of the input image like a digital camera. The second step uses microscopic slides for the Ichthyophthiriusmultifiliis, and the third portion uses the slides to identify Ich (ichthyophthiriasis or white spots). This method is used for extracting the characteristics from microscopic pictures after removing the noise and background constraints. For feature extraction, ORB (Oriented Fast and Rotated Brief) is used, and finally, it employs Logistic Regression Machine Learning Algorithm and Ant Colony Optimization (ACO) to classify Ich-infected and Non-Ich-infected fish images and get classification accuracy. Using PYTHON on microscopic samples for the causal, the suggested solution obtained an accuracy of 92.8 percent.

Liu et al.^8^ set out to create a WSSV detection system that was both extremely sensitive and fully automated, with a specific focus on ICP11 (the most highly expressed WSSV protein). The detection limit for ICP11 protein using IMR was about 2 × 103 ng/ml, and the linear dynamic range of the assay was 0.1–1 × 106 ng/ml, after the magnetic reagents were characterised. IMR signals from shrimp with low WSSV genome copy counts were successfully identified in tests of ICP11 protein in pleopod protein lysates from healthy and WSSV-infected shrimp. As a result, we came to the conclusion that the WSSV may be detected using an IMR assay that targets ICP11.

Image preprocessing and segmentation are performed in the elementary phase of the two phase system, which was developed by Ahmed et al.^9^. Feature extraction and illness classification using the Support Vector Machine (SVM) technique of machine learning with a kernel function will take place in the second phase. Fish illness is studied using an SVM model on a salmon fish picture dataset that incorporates the first phase results. A 91.42 and a 94.12 percent accuracy rates were achieved when the implementation was done with and without picture augmentation.

To detect the White Spots seen in shrimp tissues, Lakshmi et al.^10^ used morphological operator and transformation techniques in image processing. White Spot Syndrome Virus (WSSV) has been identified as the virus that causes the disease, and it is most commonly seen in shrimp farming regions, reducing the country's GDP by a significant amount. This approach can be used to block the export of faulty cartons.

According to their records, Shrimp from the Mekong Delta accounts for more than 80% of Vietnam's total output, making it one of the country’s top five shrimp exporters globally. An increasing number of shrimp farming regions are at risk from illness, according to Duong-Trung et al.^11,12^. In the event of a disease epidemic, early detection and clinical healing procedures may lead to on-site diagnoses, immediate service recommendations, and first-line treatments. The authors make a significant contribution by using transfer learning to train numerous deep convolutional neural networks and investigating six commonly reported shrimp ailments. Ninety-two percent precision in categorization has been achieved. Shrimp experts, computer scientists, treatment agencies, and politicians all focused their emphasis on developing preventative measures against shrimp illnesses over the course of the research.

Shrimps, according to Ahmed et al.^13^ are one of the most important animals in aquaculture. Shrimp output has increased steadily during the last fifty years all over the world. Many nations want to grow their CAGR and output to 5.5 tonnes in 2021. Shrimp production, however, is still beset by significant issues, such as feed quality and availability, production costs, seed quality, and illnesses. These issues must be addressed. Aquaculture is home to a variety of illnesses, including blackgill and white spot disease. If infections are detected too late, they might spread to other shrimp, resulting in the extinction of the whole shrimp species. Two forms of shrimp diseases (white spot illness and blackgill) are detected, and infected shrimp are distinguished from healthy shrimp using an effective new approach called transfer learning models. In order to diagnose shrimp sickness, the major goal was to employ the most accurate transfer learning model. The greatest degree of validation accuracy was achieved through the use of five different kinds of transfer learning. In experiment one, MobileNetV1 was 95% accurate, while in experiment two, it was 92.55% accurate. Infected fish can be recognized by combining perfect image processing and machine learning algorithms. In this case, the task was divided into two sections. Preprocessing and segmentation of images are dealt with in the first section, and then feature extraction is performed using the Support Vector Machine (SVM) machine learning technique and a kernel function, both of which are very accurate (accuracy of 91.42% and 94.94% respectively). Indeed, different technologies are being deployed to mitigate the spread and enhance the control of diseases as confirmed in various studies^1,2,14,15^. The COVID-19 crisis has affected many shrimp farmers both economically and otherwise. The spread of white spot disease among the farmers worsens their conditions which have been badly affected by COVID-19. The existing models are not much efficient and capable for predicting the White Spot disease. Hence these facts motivated the authors to develop the Ensemble learning model for purposed of predicting this disease. The proposed model would be helpful in helping to stem the spread of white spot disease.

### Definitions

- Disease pattern
  Infection may cause illness or not, depending on the features and concerns that have not yet been fully understood, but in the event of species tolerance and environmental triggers, a sufficient acceptable infection dosage provides ample time before mortality in the absence of disease. There may be a tolerance for sickness in animals with high haemolymph virions concentrations, but this does not necessarily mean that the animals are immune to the disease. There are certain vulnerable species that are unaffected by large virus loads^16^.
- Transmission mechanisms
  Vertical or horizontal spread of infection is possible. When an animal eats diseased tissue, it transmits the disease (e.g. cannibalism, predation, etc.). It's also possible to get it through water. From healthy animals, as well, the spread of illness might take place. Animals that are deceased or otherwise incapacitated can also act as a vehicle for the spread of illness^17^.
- Prevalence
  Wild populations afflicted with a given illness can have prevalence rates as low as 1% whereas captive populations can have prevalence rates as high as 100%.
- Geographical distribution
  As far as we know, WSD was first discovered in the Middle East. China (People's Republic), Japan, Korea, Southeast Asia, the Indian Continent, the Mediterranean and the Americas are all examples of crustaceans that exhibit this trait. There are also WSD-free zones and compartments in these areas^18^.
- Mortality and morbidity
  Penaeid shrimp are all susceptible to illness, resulting in high death rates. Several species of crustaceans and prawns, as well as lobsters, are susceptible to infection, although the severity of the illness and death that ensue from infection varies widely. In the absence of medical research, it is common to observe severe infections in various decapods.
- Environmental factors
  An outbreak of illness may occur as a result of stresses like as sudden changes in salinity. WSD outbreaks are more likely to occur when the average water temperature is below 30 °C, which is why it is important to monitor water temperature.
- Control and prevention
  A higher percentage of 'vaccinated' shrimp and crayfish make it out of the WSSV challenge, despite the fact that the cause is yet unclear. Oral injection of bacterially produced VP28 dsRNA has been shown to protect shrimp against WSSV infection in recent investigations. As a result, protection has been available in the form of immunisation or RNAi thus far^19^.

## Materials and methods

### Dataset

This dataset is available in Mendeley and it is useful as the baseline data for analyzing the changes of shrimp farming in the coming years in Bangladesh. For further statistical analysis, it may be used to produce nominal/categorical/dependent variables by reclassifying the many variables. This dataset serves consists of following variable for conducting the research:

- Characteristics of the site or farm (A former exploitation of the land, Size of the pond used in manufacturing (hA), Typical Soil Composition, Common Canal Depth, Standardized farm width and Cultural Norms)
- An External Factor (Salinity, Temperature and pH)
- Anatomy of an Illness (Historical occurrence of WSD & Detection of Virus (current culture))
- Variables related to site/farm management (Owner-run farm, Application of fertilizer, Abuse of Chemicals (pond preparation), Substitution of Chemicals (water treatment), Aeration system, Gher being dried following harvest, Technique for Draining Sludge, Time it takes to drain the sludge, Keep the dikes in good shape by repairing them as necessary and The Fallow Time (days))
- Factors under administration (Water management)
- Controllable factors in administration (Culture management)
- Indicators of Management Success (Feed management)
- Situational factors in management (Biosecurity management)

The data set was evaluated using multivariate statistical methods to determine the causes of WSD, and the results were reported in a scientific paper. Policymakers and development groups in Bangladesh used the findings to mitigate the effects of WSD on the country's shrimp farming industry^20^. The data set's potential applications & vast number of variables in this dataset makes it suitable for additional statistical investigation; some of these variables can be recoded to create new nominal, categorical, or dependent ones. Each of the 233 farms' unique GPS coordinates is included in the dataset, allowing for future spatial and longitudinal analysis. The data collection posted in Mendeley has served as a starting point for future studies of shrimp aquaculture in Bangladesh^21^.

### Methods

- Data Pre-processing

Data preprocessing is a necessary step for machine learning and deep learning tasks, and it is the basis of algorithm development, ML model training, and computer vision or image processing systems^22^. Basically it performs data quality assessment as there are numerous data anomalies in the datasets. Missing data is filled in, and inaccurate or irrelevant data is repaired or removed from a data collection via data cleaning. To guarantee that our data is ready to be used in the future, data cleaning is the most critical phase of preparation.

- Missing values

The most typical objective of data preparation techniques is to replace null values since it allows us to deal with a whole dataset. Values or data that are not saved or missing from the dataset are referred to as missing values. We imported SimpleImputer from the sklearn package to carry out substituting null values as part of data preparation. Imputing or expressing missing values is simplified with the Simple Imputer class^23^.

- Outliers data

An outlier is a value that differs considerably from the rest of the data. Outliers are data points that differ from the rest. They may occur due to variability in the dimension or sometimes may point out experimental errors. As a way to deal with outliers, we used the multivariate technique, which builds an accurate prediction model out of every piece of data and eliminates any occurrences that have error values over a certain threshold^24^.

#### Random forest

Machine learning is very rich in algorithms to train the model and predict the results. Broadly the algorithms have been categorized into supervised and unsupervised machine learning algorithms^25^. Random Forest is one of the most popular machine learning algorithms which comes under supervised machine learning category that is used widely in classification and regression. Random Forest technique is a very easy to use among machine learning algorithm and it is capable of producing great results even in the absence of hyper-parameter tuning. Due to its simplicity and diversity as can be used either in classification or in regression tasks too, it is more demanding in industries. The working of Random Forest gets involved in decision tree building with different samples and later finalizes the results on the basis of majority in case of classification and takes average in case of regression^26^.

**Figure 1:**
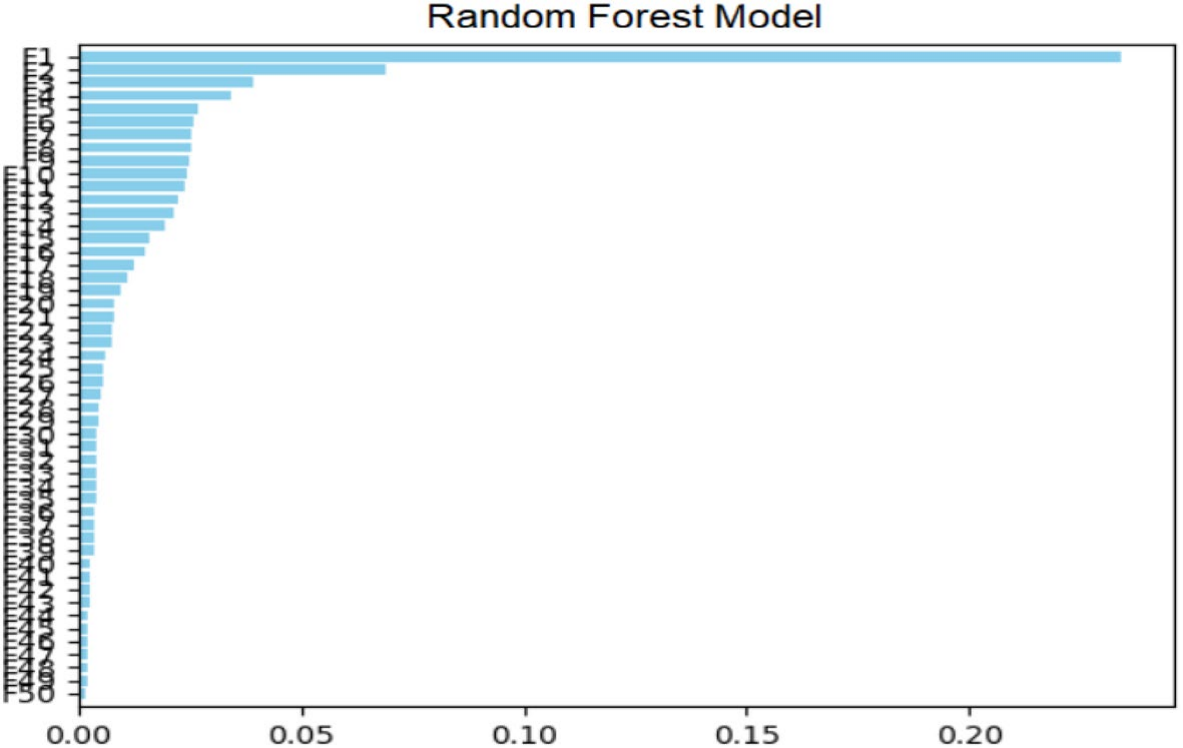
Predictor importance in random forest.

Figure 1 represents predictor importance in Random Forest for current problem. In this figure, X axis labels the predicator value range and Y-axis represent the different predicators used for predicting current disease. The training phase of a Random Forest method requires the adjustment of three primary hyperparameters. They are the node size, the number of trees, and the number of characteristics sampled. After that, the Random Forest classifier may be utilized to address any sort of regression or classification issue.

The steps of the actual Random Forest Algorithm are as follows:

Step 1. Pick at random from a dataset or training set.
Step 2. For each piece of training data, this algorithm will create a decision tree.
Step 3. The decision tree will be averaged, and that's how votes will be cast.
Step 4. Last but not least, take the forecast outcome that received the most votes and use that one.

Each tree in the ensemble of the Random Forest method is built using a data sample selected from the training set with replacement, known as the bootstrap sample. Afterwards, feature bagging is used to introduce yet another random event into the dataset, increasing the variety of inputs while decreasing the correlation between different decision trees^27^. The manner in which the forecast is arrived at varies with the nature of the underlying problem. Individual decision trees will be averaged for a regression work, while the most common categorical variable will win out in a classification task and be used to determine the projected class^28^. When the cross-validation process is complete, the prediction is locked. Major Obstacles are being faced in applications of this model as given below:

- Slow procedure: As a result of processing data for each individual decision tree, Random Forest algorithms can produce more accurate forecasts, but they can be time-consuming to analyze huge datasets.
- Takes more effort and supplies because: More storage space is needed for Random Forests since they analyze bigger datasets.
- Greater in complexity: A single decision tree's forecast is more straightforward to decipher than that of a whole forest of trees.

#### CHAID

In 1980, Kass came up with the idea for this algorithm. This method uses the chi-square statistic as its basis. If the outcome of a Chi-square test is between zero and one, the test returns a probability value. The chi-square value is close to 0 when there is a considerable difference between the two classes being compared. There is no substantial difference between two classes that are being compared if the chi-square result suggests a value close to 1.

Creating CHAID tree consists of the following stages^29^:

1. The root node should contain all of the training information.
2. The kind of dependent variable should guide which independent variables to test for statistical significance.
3. When subsetting prescribed dataset, researchers often choose the variable with the lowest p-value from the statistical tests as the dividing line. (We may modify the alpha significance level using the Bonferroni adjustment. When there are more than two groups in a categorical predictor variable, we can combine the ones that are not significant.
4. Step 3 is repeated with the different data sets using the independent variables until
  - It's either impossible to find any more information about the dependent variables or they're not statistically significant at the alpha level.
  - In this case, we've reached the endpoint.
5. The tree's leaf nodes should be used to generate business rules.

**Figure 2:**
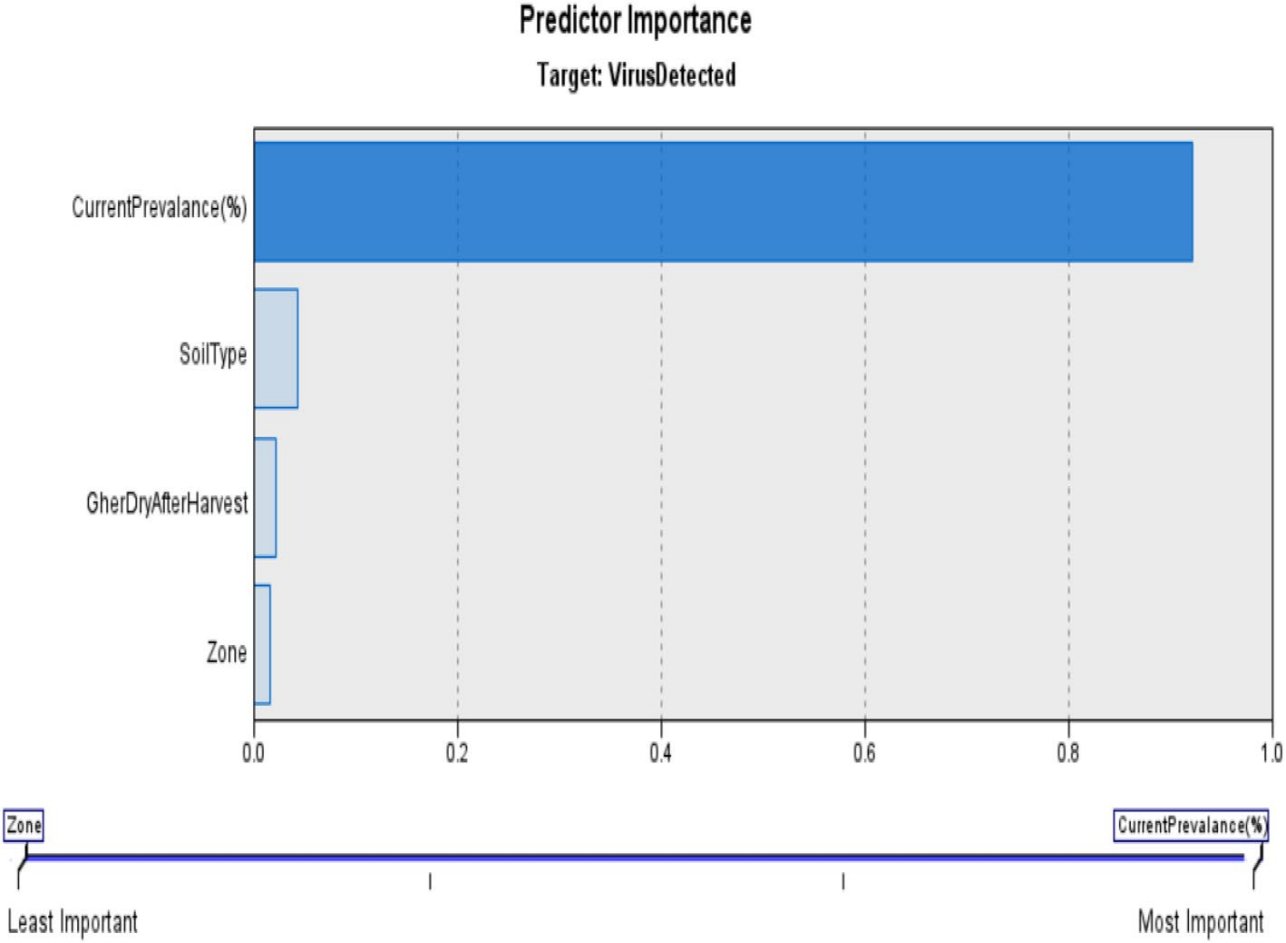
Predictor importance in CHAID.

Figure 2 represents predictor importance in CHAID for current problem.

CHAID initially runs a chi-square independence test on the crosstabulations displaying the correlations between the various inputs and the outputs. CHAID will choose the most significant input field if more than one of these relations is statistically significant (smallest p value). When there are more than two categories in an input, they are compared, and any that don't significantly alter the output are collapsed. To do this, it combine together the two groups that vary the least^30^. Once all remaining categories have a difference at the threshold you set for testing, the merging procedure will end. Any two categories in a nominal input field can be combined, however in an ordinal set, only adjacent categories can be combined.

Even though CHAID was intended to analyze non-metric and non-ordinal data that standard multivariate analyses cannot handle, it still have some restrictions:

- Any information collected must be of an ordinal, nominal, or interval kind, and not metric. There must not be more than 15 values for any variable. All metric variables and those with more than 15 levels must be recoded to have no more than 15 distinct categories^31^.
- Identifying a "response" or dependent variable is necessary. This corresponds to the clustering variable in discriminant analysis. The sample will be divided by CHAID so that the differences (variance) between the groups is maximized. Without a dependent variable of this type, CHAID cannot be executed. A new perspective may be obtained by running CHAID using segments created by a clustering algorithm as the dependent variables^32^.
- CHAID is not able to handle zeros or out-of-order codes (for instance, it cannot be skipped from a code "3" to a code "6"). There's a chance that this will increase the amount of time spent recoding information^33^.

#### Proposed ENRFCH model for prediction

In this research work, the proposed method titled as ENRFCH (**En**semble **Random Forest**&**CH**AID) method has been designed for handling the limitations of decision tree models. However, regardless of the specifics, Decision Trees share the same limitations as any other model.

- Decision Trees' most serious shortcoming is its susceptibility to overfitting. Since a leaf node may be created for each goal value in the training data, decision trees tend to overfit. As a matter of fact, in sklearn, it is how the Decision Tree Classifier/Regressor is often configured. Simple Decision Trees are commonly swapped out for their more complicated relatives, the ensemble approaches, because of the requirement to modify some hyperparameters like max features, min samples split, min samples leaf, and max depth.
- The decision-making process of a Decision Tree is also locally optimized, or greedy, which simply means that it does not plan ahead when determining how to split at a specific node. Instead, splits are formed to minimize or maximize the selected splitting (selection) criterion, which we'll get into later (gini or entropy for classification, MSE or MAE for regression).
- However, Decision Trees have trouble with classification problems involving unbalanced classes due to the greedy nature of splitting. It is the job of the tree, at each fork, to determine how optimally to divide up classes across the two resulting nodes. Prediction of the minority class is considerably less likely than it should be, if any nodes predict it at all, when one class has extremely low representation (the minority class), because many of those observations might be buried in the nodes of the majority class.

Ensemble means a mix of multiple models. Hence in the case of ensembles a collection of models is used to make predictions rather than an individual model. Ensemble uses two methods basically one is bagging and another is boosting^34^. All decision trees can be combined in order to produce a generic outcome by using bagging as the only method. It is a sampling approach with a substitution of the sampled data. Subsets of data from the original dataset must be created using this method, and the size of the subsets must match the original dataset^35^. A clear and impartial picture of the distribution may be obtained by using these subsets or bags in Bagging (or Bootstrap Aggregating) approach. Boosting is a series of iterations aimed at improving the accuracy of the final model by correcting the flaws introduced by the preceding iteration^36^.

Steps of ENRFCH model for prediction

There are following steps to be performed:

Step 1. Initially create a subset from the original dataset.
Step 2. All data points are given equal weights initially.
Step 3. Next create Random Forest model on this subset.
Step 4. Apply Random Forest model to make predictions on the whole dataset. Further calculate the errors using the actual values and predicted values. The incorrectly predicted observations are given higher weights.
Step 5. The CHAID model is created and predictions are made on the dataset and try to correct the errors from the previous model through applying specified cost function.
Step 6. The weighted mean of all the models is the final model.

When a new model is created, it relies on what came before. Each model may or may not work well on all of the data, but there is a good possibility that it will work well on some of the data. This is why the algorithm boosts weak classifiers into strong classifiers^37^. As a result, each model really enhances the group's overall performance. As opposed to a loss function, which often provides less control and does not fundamentally correlate with real-world applications, gradient boosting based ENRFCH model allows one to optimize a user-specified cost function.

### Informed consent

It is to confirm that the patients are not required to give Informed consent statement for this study as the dataset is available on mendeley data website for open access.

## Results

### Experimental Setup

For this experiment, the authors performed the following steps to initialize the defined parameters for executing the optimal final model in Table 1 as:

**Table 1:** Defined parameters.

Parameter	Value
is_classification	True
enable_gpus	True
seed	False
accuracy	7
time	2
interpretability	8
num_prediction_periods	None
num_gap_periods	None
is_timeseries	False
is_image	False

These Accuracy, Time, and Interpretability settings map to the following internal configuration of this experiment in Table 2 as given below:

**Table 2:** Internal parameters.

Internal parameter	Value
Data filtered	False
Number of feature engineering iterations	10
Number of models trained per iteration	8
Early stopping rounds	5
Monotonicity constraint	True
Number of model tuning model combinations	9
Number of base learners in ensemble	3
Time column	[OFF]

### Performance evaluation

The proposed work consists of following models which are used for the prediction of WSD:

- Random Forest model
- CHAID model
- Proposed ensemble model

The performance of these algorithms are being evaluated on based of following three factors as given below:

- **Accuracy**: One way to rank classification algorithms is by how well they perform^38^. Our model's accuracy may be loosely defined as the percentage of correct predictions it made. There is a strict definition of accuracy that goes like this:

1 $$Accuracy = \frac{No \; of \;corrected \;predictions}{{Total \; no \; of \; Predications \; made}}$$

Accuracy can also be determined in terms of positive and negative results for binary categorization.

2 $$Accuracy = \frac{TP + TF}{{TN + TP + FP + FN}}$$

In which False positives (FP) and false negatives (FN) are defined as the opposites of true positives (TP) and true negatives (TN).

- **AUC (Area Under the ROC Curve):** AUC stands for "Area under the ROC Curve". The area under the ROC curve (think integral calculus) from (0,0) to (1,1) is what AUC measures (1,1). The area under the curve (AUC) measures the overall effectiveness of a classification system across all possible cutoff points^39^. One possible interpretation of AUC is as the likelihood of a good example being ranked higher than a negative example by the model. For these two reasons, AUC is preferable as it is independent of scale. The accuracy of ranking forecasts is what this metric focuses on rather than the actual worth of the predictions themselves^40^. Second, the accuracy of a classification is irrelevant to the AUC calculation. Quality of predictions is measured independently of the categorization criterion used.
- **Gini index:** The Gini Coefficient is used to measure the accuracy of Binary Classifier Models in the field of machine learning. When calculated, the Gini Coefficient can take on a value between zero and one. As the Gini coefficient rises, so does the quality of the model being evaluated. This measurement is frequently employed to assess the accuracy and robustness of various models^41^.

**Figure 3:**
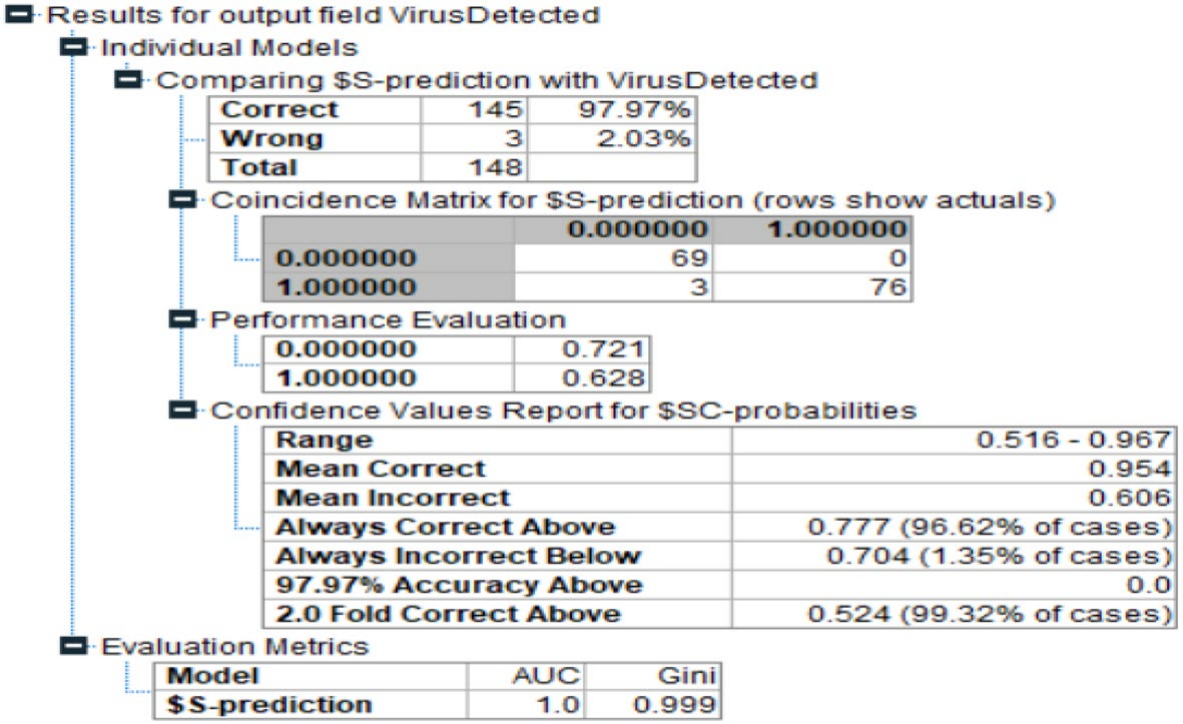
Accuracy in Random forest.

In the first phase, Random Forest model for the current problem is implemented. Figure 3 demonstrates the results generated from the Random Forest model. The accuracy level is achieved up to 97.97%.AUC and Gini index of this model are 1.0 and 0.999 respectively.

**Figure 4:**
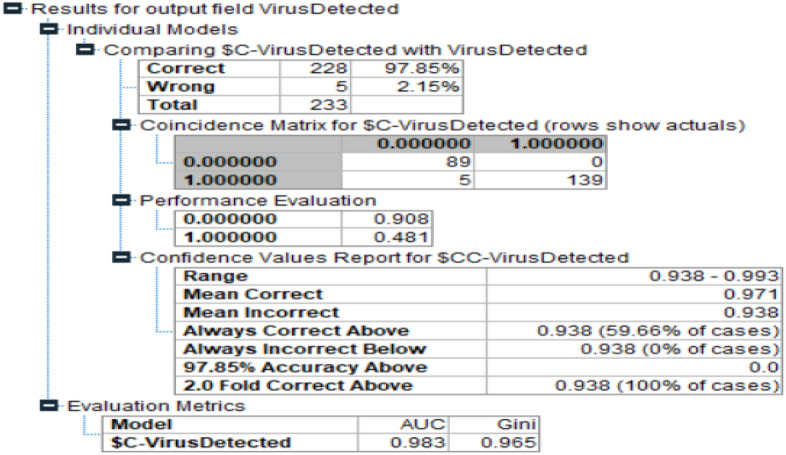
Accuracy in CHAID.

In the second phase, the authors have implemented the CHAID model for the current problem. Figure 4 demonstrates the results generated by this model. The accuracy level achieved upto 97.85%. The AUC value of this model is 0.985 and GINI index is 0.965.

**Figure 5:**
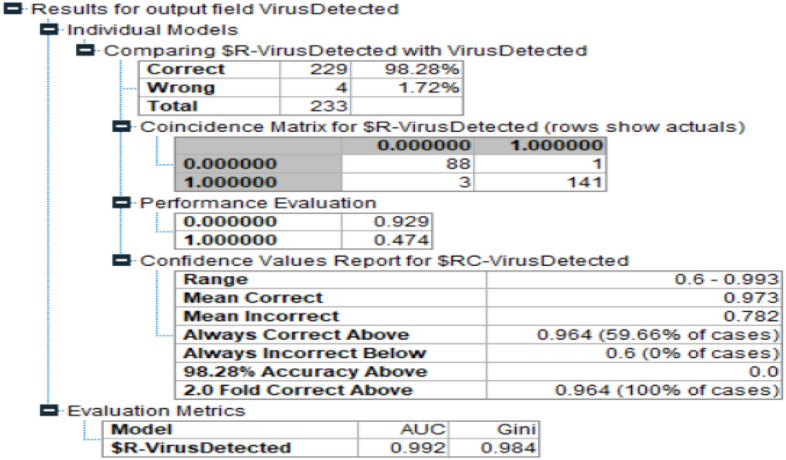
Accuracy of the proposed model.

Lastly in the final phase the authors implemented the proposed ENRFCH model for the current problem. Figure 5 demonstrates the results generated by this model. The accuracy level achieved by this model is 98.28%. The AUC value of this model is 0.992 and GINI index is 0.984.

Hence as per the accuracy levels achieved in the proposed model using bootstrapping is predicting more accurately. Table 3 below gives the summarized results of the performance comparison:

**Table 3:** Model performance comparison.

Sr. No.	Model	Accuracy level	AUC	GINI index
1	Random Forest	97.7	1.0	1.0
2	CHAID	97.85	0.985	0.965
3	Proposed Model	98.28	0.992	0.984

The proposed method uses machine learning techniques (Random Forest & CHAID) and provides an accurate sys-tem that can identify white spots disease with an accuracy of 98.28% which is remarkable performance in comparison with the previous methods.

Table 4 demonstrated the performance of proposed model in terms of other performance metrics except of Accuracy, AUC & Gini values. These parameters defines the reliability and flexibility of the proposed model. To prevent the model from becoming overly specific to its training data, this proposed method attempts to pick the point where performance on the test dataset begins to decline automatically. White Spot Syndrome Virus (WSSV), the causative virus of the disease is found in most shrimp farming areas of the coastal areas, and has the potential to cause major economic losses specifically to the shrimp farming industry which also results in decay of GDP of any country (Supplementary information).

**Table 4:** Performance of proposed model.

Scorer	Better score is	Final ensemble scores on validation (internal or external holdout(s)) data	Final ensemble standard deviation on validation (internal or external holdout(s)) data
AUCPR	Higher	0.9902729	0.008556496
F05	Higher	0.9933036	0.005006364
F1	Higher	0.984936	0.01302901
F2	Higher	0.9758161	0.01707978
FDR	Lower	0.001194593	0.003189716
FNR	Lower	0.02554489	0.0203418
FOR	Lower	0.04246907	0.03906844
FPR	Lower	0.004822281	0.008269834
LOGLOSS	Lower	0.4509245	0.249029
MACROAUC	Higher	0.9884023	0.01103452
MACROF1	Higher	0.9844569	0.01209231
MACROMCC	Higher	0.9625525	0.02915984
MCC	Higher	0.9564286	0.03188604
NPV	Higher	0.9570081	0.03895281
PRECISION	Higher	0.9979197	0.003722273
RECALL	Higher	0.9725892	0.02315772
TNR	Higher	0.9972702	0.005393785

## Concussion and future work

The study presented solution for prediction of White Spot Disease among shrimp farmers using machine learning algorithms. The biggest problem with random forest is that it can become too inefficient for real-time forecasts if too many trees are used. Typically, these algorithms are quick to train but take a long time to make predictions when training is complete. The proposed method uses machine learning techniques (Random Forest & CHAID) and provides an accurate system that can identify white spots disease with an accuracy of 98.28% which is remarkable performance in comparison with the previous methods. Hence the proposed method is highly recommended for early prediction of WSSV in aquaculture. The proposed methodology shows the efficient method to find the white spots on shrimp farmers and predicts the likelihood using a smoothened dataset through data pre-processing techniques. Scientists have discovered that the virus poses a risk not only to shrimp but to a wide variety of other crustaceans found in both saltwater and freshwater environments. Too far, no effective preventative treatment strategies are available for viral infections in shrimp and other crustaceans. Due to present aquaculture techniques and the extensive host range of WSSV, intervention efforts including immunization against this virus would be important to rescue and safeguard shrimp farming. Several successes have been obtained in the search of new vaccines for WSSV. DNA vaccination, recombinant vaccines, oral vaccination approaches and gene therapy are some of the key areas of interest for scientists and researchers. The future work would focus on development of Artificial intelligence and internet of things (IoT) driven smart devices to assist in helping shrimp farmers to check their white spot disease status especially after COVID-19.

## Supplementary Information

Supplementary Information.

## Data Availability

The datasets used during the current study available from the corresponding author on reasonable request. The dataset may be downloaded from following web line as given below: https://data.mendeley.com/datasets/nz96v5spbf/2.
